# Hydrophobic complementarity-determining region 3 (CDR3) sequences elucidate the cardiotoxic effects of immune checkpoint inhibitors

**DOI:** 10.21203/rs.3.rs-8320867/v1

**Published:** 2025-12-16

**Authors:** Shoiab Bukhari, Rajat Mohindra, Matthieu Paiola, Carly Tymm, Robert Winchester, Aditi Guha, Laura Tang, Sanjay Bansal, Brian S. Henick, G. Scott Chandler, Adam Mor

**Affiliations:** Columbia University Medical Center; F. Hoffmann-La Roche; Columbia University Medical Center; Columbia University Medical Center; Columbia University Medical Center; Genentech, Inc; Genentech, Inc; Genentech, Inc; Columbia University Medical Center; F. Hoffmann-La Roche; Columbia University Medical Center

**Keywords:** Cardiotoxicity, myocarditis, effector T cells, immune-related adverse events, perforin 1, T cell receptor, CDR3, hydrophobic TCR

## Abstract

Immune checkpoint inhibitors (ICIs) have significantly changed cancer treatment, demonstrating strong efficacy across multiple cancers. However, their use also carries the risk of serious immune-related side effects (irAEs), especially cardiotoxicity. To understand how these adverse effects occur, we studied peripheral blood mononuclear cells and T cells taken from the heart tissue of cancer patients who experienced cardiotoxicity during ICI therapy. Using spectral flow cytometry, single-cell RNA sequencing, and T cell receptor (TCR) sequencing, we found key differences in the immune profiles of affected patients. Those with cardiotoxicity had a noticeable increase in circulating CD4 + FOXP3 + and CD8 + PRF1 + T cells at disease onset. Our results also show that effector CD8 T cells are present in the heart tissue and pericardial fluid of patients with myocarditis, highlighting their role in starting the disease. TCR sequencing revealed expansions of CD8 GZMK + GZMA + and CD8 PRF1 + GZMA + T cells in myocarditis patients, along with increased activation markers CD69 and KLRG1, supporting the idea that specific cytotoxic CD8 T cell groups promote inflammation. Notably, we also found that T cells from patients with irAE myocarditis have shorter TCR CDR3 sequences, with a higher proportion of hydrophobic residues. This discovery suggests a new mechanism for TCR involvement in irAE myocarditis, focusing on T cell activation through the TCR’s functional promiscuity, which relies more on TCR-MHC interactions than on specific peptide features. Overall, this research provides a foundation for new strategies targeting TCR physical properties to reduce risks and develop more precise therapies for vulnerable patients.

## INTRODUCTION

Immune checkpoint inhibitors (ICIs) have significantly improved outcomes for patients with cancer (1–5). However, they can also lead to immune-related adverse events (irAEs), including cardiotoxicity. This ICI-related cardiotoxicity is a serious condition, with a mortality rate of up to 50% for myocarditis (6), and often necessitates the permanent discontinuation of ICI therapy (6–8). ICIs enhance the immune system’s ability to combat cancer by targeting immune checkpoints that regulate immune responses. While effective against tumors, ICIs may inadvertently trigger autoimmune-like responses, resulting in inflammation in various organs, including the heart (9–11). The onset of irAE cardiotoxicity, particularly myocarditis, is complex and involves T cell activation, cytokine release, and potential autoantibody formation. These mechanisms result in heart muscle inflammation, which can lead to chest pain and shortness of breath. In half of the cases, myocarditis can lead to heart failure, arrhythmias, and death (6–8). Diagnosing irAE myocarditis can be difficult due to symptom overlap with other cardiac conditions. Confirmation generally requires clinical evaluation, cardiac biomarkers, electrocardiograms, imaging tests such as cardiac MRI, and occasionally an endomyocardial biopsy.

There is no consensus on how to treat these patients. New experimental, targeted, biologically informed therapies, such as abatacept and ruxolitinib, are under investigation (12). Timely diagnosis and intervention are essential for achieving optimal patient outcomes. Ongoing research aims to clarify the complex mechanisms of irAE myocarditis, paving the way for improved diagnostic tools, prognostic markers, risk mitigation strategies, and personalized therapies for intervention. This understanding is vital for minimizing the risks associated with ICI therapy while maximizing its advantages for cancer treatment at both early and advanced disease stages.

Previous research has highlighted the roles of macrophages, T helper 17 (TH17) cells, B cells, autoantibodies, and possibly α-myosin-specific T cells in the development of irAE myocarditis (1, 2, 13). Blum et al. identified cardiac-expanded T cells in circulation (14). However, there remains a need for a deeper understanding of T cell involvement in this condition and specific strategies to mitigate it. To address these critical gaps, we conducted a comprehensive investigation using single-cell RNA and T cell receptor (TCR) sequencing to characterize T cell populations in patients experiencing ICI-induced cardiotoxicity. We aimed to test the hypothesis that not only specific subsets of T cells drive irAE cardiotoxicity, but also the characteristics of the TCR CDR3 sequences mechanistically contribute to the pathogenesis of this condition.

## METHODS

### General reagents

RPMI 1640 medium, Dulbecco’s PBS, and FBS were purchased from Life Technologies.

### Patient selection

The initial cohort included blood samples from ten patients with ICI-induced cardiotoxicity, specifically myocarditis, heart failure, and AV block, from various completed clinical trials using atezolizumab, an anti-PD-L1 therapy, either as monotherapy or in combination with other treatments across different tumor types. The study investigators diagnosed and reported ICI cardiotoxicity events based on their clinical assessments, guidance provided in the study protocols, and guidelines from oncology medical societies. Blood samples were also collected at multiple time points from five patients who did not experience ICI-related cardiotoxicity during the study period and were selected as individual controls. The inclusion criteria for the clinical trials required patients to have histologically confirmed cancer, to be treatment-naive regarding immunotherapy, and to have a planned immediate initiation of immunotherapy. Exclusion criteria included active infection, a history of autoimmune disease, use of immunosuppressive therapy, current pneumonitis or pulmonary fibrosis, and recent live-virus vaccinations. These patients consented to future exploratory biomarker analysis as part of their enrollment in the atezolizumab study trials. Additionally, we identified two patients diagnosed with myocarditis following atezolizumab therapy as standard care at the oncology center of Columbia University Medical Center. Pericardial fluid was collected from these patients. A public single-cell RNA and TCR sequencing dataset of myocardial T cells from an additional fourteen patients with ICI myocarditis was used to validate the results. Blood samples from four patients with ICI arthritis and three healthy controls were also used.

We analyzed the treatment regimens and durations for all participants. Information regarding the occurrence, diagnosis, management, and outcomes of irAEs was also reviewed. Blood samples were collected from patients before starting atezolizumab as part of the clinical trial protocol and standard ICI therapy. For those with ICI-induced cardiotoxicity, blood samples were taken at baseline and during treatment. Each patient provided 10 mL of blood in EDTA vacutainers before treatment began. For some patients, an additional 10 mL of peripheral blood was drawn while on ICI treatment; this time point could be close to the occurrence of ICI cardiotoxicity. In the control group, the timing of the second blood draw was aligned with the interval between therapy initiation and blood collection for the irAE group.

### Flow cytometry and antibodies

For protein expression analysis following isolation, cells were collected and stained with the antibodies listed below in a comprehensive T cell panel for surface protein expression: CD278, IL-17RA, TIM-3, CD45RA, CTLA-4, LAG-3, KLRG1, KLRB1 (CD161), KLRF1, CD5, CD56, CD57, CD69, CD185 (CXCR5), CD183 (CXCR3), CD95, CD194 (CCR4), CD4, PD-1, PD-L1, HLA-DR, -DP, -DQ, CD28, CD27, TCR alpha/beta, TCR Valpha7.2, CD196 (CCR6), CD8, SLAMF6, CD103, CD127, CD15, CD14, CD19, CD16, IL-17RB, IL-23R, CD62L, CD294 (CRTH), CD25 (IL-2RA), IgG4, IFN-γ, IL-17A, IL-21, CD185, CD183, CD194, CD45RA, CD3, CD62L, CD8, TNFα, IL-21, CD45RA, GM-CSF, and PD-1. Each analysis aimed to explore unanticipated phenotypes using dimensional reduction algorithms. Dead cells were excluded from the analysis using Zombie-UV (Biolegend). Doublets and double-positive CD4/CD8 cells were removed through sequential gating. Flow cytometry acquisition was performed using an Aurora 5 laser cytometer. Data was analyzed utilizing FlowJo 10.1r7 and GraphPad Prism 9.

### Isolation and preparation of cells for RNA sequencing

Peripheral blood mononuclear cells (PBMC) were isolated using the Ficoll gradient. Cell concentration, singularity, and viability were verified using a hemocytometer before submission for scRNA-Seq (10X Genomics). Cells from various patients were hashtagged using a multiplexing strategy to enable individual sample identification. The hashtagged cells were then pooled and suspended before being barcoded with gel beads and partitioning oil. The cells were loaded onto the 10x Genomics Chromium Chip to produce single-cell Gel Beads-in-Emulsion. (GEMs). Captured cells were lysed, and their transcripts were barcoded via reverse transcription within individual GEMs. The resulting cDNA, along with cell barcodes, was then amplified by PCR. Using the 5’ Library Kits (PN-1000165, PN-1000020), scRNA-seq libraries were constructed, while scTCR-seq libraries were created with the V(D)J Enrichment Kits for Human T Cells (PN-1000016, PN-1000005). Following the manufacturer’s recommendations, a single-cell sequencing library was generated using a segmentation-based approach. The cDNA libraries were then sequenced using Illumina 150 bp paired-end sequencing.

### RNA-sequencing data analysis

The raw sequencing data were processed using the Cell Ranger pipeline (v3.0.1, 10x Genomics), which included steps such as demultiplexing, genome alignment to GRCh38, barcode counting, and unique molecular identifier (UMI) counting. The resulting gene-barcode matrix of UMI counts was then analyzed with Seurat (v4.3.0.1) for quality control, normalization, dimensional reduction, batch effect removal, clustering, and visualization. The count matrix was log-normalized, and the top 3,000 most variable genes were selected for dimensional reduction. All samples were integrated using canonical correlation analysis (CCA) to remove batch effects. The integrated matrix was then scaled, and the top 13 dimensions from principal component analysis (PCA) were used for uniform manifold approximation and projection (UMAP). The resolution was set to 0.3 to identify significant cell types of PBMCs. Cell identities were manually determined by selecting curated marker gene sets (Sup. Table 1) for each subset from publicly available scRNA-seq studies. The Human Primary Cell Atlas was used to compare the transcriptomes of each cell cluster. Marker genes for each cluster were visualized by plotting heatmaps using Phantasus (https://ctlab.itmo.ru/phantasus/), facilitating the assignment of cell types. Graphs, including bar, box, violin, and scatter plots, were generated using Prism 9 (GraphPad Software). Signature enrichment scoring was conducted by calculating z-scores relative to naive T cells, when indicated, or by using the scoring method described by Tirosh et al. Gene Ontology analysis. The GSE228597 dataset (heart tissue) was reanalyzed using the Scanpy package, deploying a standard pipeline for quality control and analysis (https://scanpy.readthedocs.io/en/stable/tutorials/index.html).

#### CDR3 sequences analysis and hydrophobicity scoring.

T cell receptor (TCR) α and β CDR3 sequences were extracted from VDJ contig files and processed single-cell TCR sequencing (scTCR-seq) data. Only productive TCR sequences were analyzed, and CDR3 clones were defined based on identical amino acid sequences. To assess the physicochemical properties of the hypervariable CDR3 region, we used the CDR3 tool package (https://github.com/caparks2/cdr3tools) to calculate average hydrophobicity scores of each CDR3 sequence. Hydrophobicity was determined using the Kyte-Doolittle scale and the Wimley-White scale, which assigns numeric values to each amino acid based on its hydrophobic character. Sequence-level hydrophobicity profiles were visualized and statistically compared between the conditions.

### Structural analysis

The structural alignment analysis of the query CDR3 amino acid sequence was performed using the TCR3d database (https://tcr3d.ibbr.umd.edu/). The TCR-pMHC I structure, identified as 7N1F, specific for a SARS-CoV-2 peptide bound to HLA-A*02, was selected as a reference based on the length of the CDR3, alignment suitability, and availability in the IMGT 3D structure database (https://www.imgt.org/3Dstructure-DB). The 7N1F PDB file was obtained from the RCSB-PDB server (https://www.rcsb.org/). To match the query CDR3 amino acid sequence found to be enriched in T cells of cardiotoxicity patients, the CDR3 of the TCR beta chain of reference structure 7N1F was mutated using PyMOL. The reference (reference-CDR3) and mutated (adjusted-CDR3) structures were prepared using Schrödinger’s Maestro package, which includes steps for structure preparation, protonation, and energy minimization with default settings. The final structures were then analyzed using the ISOLDE plugin of ChimeraX for interactive molecular dynamics simulations. Each structure was simulated for 12 hours, in addition to direct user intervention through the implementation of common tasks, such as the cis-trans change of peptide geometry and flipping of peptide orientation. The Ramachandran and rotamer quality of each residue was assessed in real-time as the models evolved. A final structural validation report, including a Ramachandran plot, was generated at the end of each simulation. The structural stability and contact profiles of the reference and mutated structures were analyzed using the Protein Contacts Atlas (https://pca.mbgroup.bio/index.html). Visualization and image generation were performed with ChimeraX. In the arthritis dataset, a hydrophobic hypervariable CDR3 sequence associated with arthritis was compared to a hydrophilic counterpart from patients without irAEs, sharing identical flanking regions.

### Quantification and statistical analysis

Unless otherwise specified, the data are presented as mean ± standard error or standard deviation of the mean. As indicated, statistical significance was determined using the Kolmogorov-Smirnov (K-S) two-sample test as for non-parametric analysis for subset variations and One-way ANOVA for assessing the differences in T cell signature scores. Statistical analyses were performed using Prism 9 (GraphPad Software). Significance was set at p = 0.05. An unpaired non-parametric test was applied wherever indicated to measure the variance between each patient at baseline and on treatment conditions. A non-parametric Spearman correlation was used to compute the correlation between signature enrichment scores, with a two-tailed p-value and a 95% confidence interval, as indicated. Our minimum effect size was 2, and we could reject the null hypothesis that the population means of the two groups are equal with an estimated power of 0.98. The FDR associated with this test is 0.01. Logistic regression analysis was performed on differentially expressed genes between the conditions, followed by ROC analysis to identify the most significant genes.

## RESULTS

### Study population and sample collection

**Figure 1:**
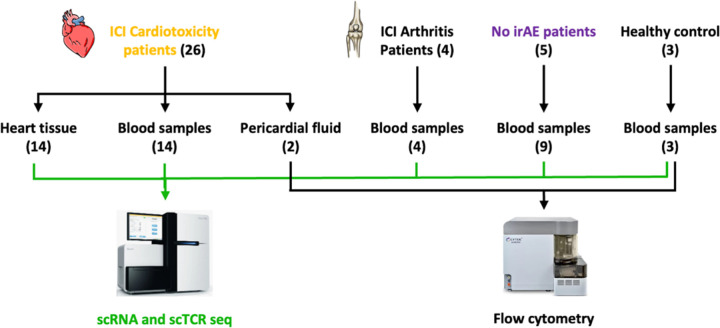
Patients’ characteristics and study design. (A) Description of the patients enrolled in the study. (B) Brief clinical characteristics.

This initial study cohort included fourteen samples from cancer patients treated with atezolizumab who developed ICI cardiotoxicity and nine samples from cancer patients treated with atezolizumab who did not develop irAE, sourced from completed clinical trials. Among these, eleven patients experienced symptomatic ICI cardiotoxicity (including myocarditis, as noted by investigators), while six patients showed no signs of ICI cardiotoxicity. Longitudinal blood samples were collected from three patients. Baseline samples were available for thirteen patients before ICI treatment. Demographic analysis revealed a mean age of 70 years for patients with cardiotoxicity (70% male) and 76 years for the cardiac irAE-free controls (40% male) (Supp. Figure 1A and 1B). Disease severity, categorized by NCI CTCAE grades (G), included G2 (n = 3), G3 (n = 6), and G4 (n = 2). Furthermore, three patients developed additional irAEs, including myositis and myasthenia gravis. To better control the experiment, we examined two pericardial fluids from cancer patients with ICI-myocarditis, four blood samples from patients with ICI arthritis, and three from healthy controls from the Columbia University Medical Center oncology practice. Peripheral blood mononuclear cells were isolated for subsequent single-cell RNA and TCR sequencing. Spectral flow cytometry was used to characterize the pericardial fluid and blood samples from three controls without irAE. The validation cohort included single-cell RNA and TCR sequencing data from an additional fourteen cancer patients treated with ICI who developed myocarditis (GSE228597) (Fig. 1).

#### Significant differences in cluster distribution between patients experiencing cardiotoxicity and controls.

**Figure 2:**
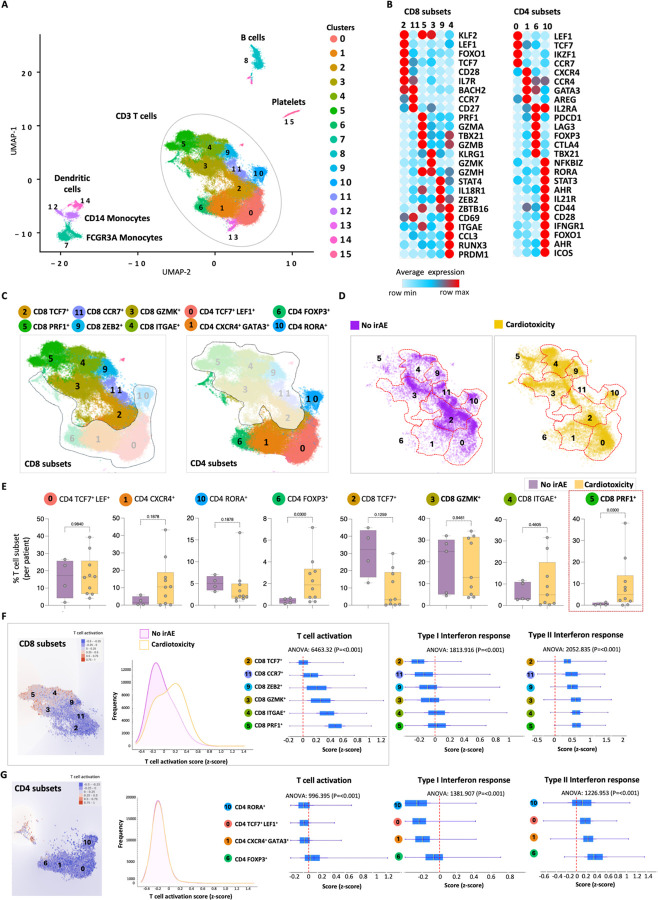
Significant differences in cluster distribution between patients experiencing cardiotoxicity and controls. (A) A UMAP plot based on scRNA sequencing of 62,959 cells reveals 15 clusters. The ellipse indicates CD3-expressing T cells. (B) Expression levels of marker genes distinguish CD4 and CD8 T cell clusters. (C) The annotation of CD4 and CD8 T cell clusters relies on the expression of marker genes. (D) The distribution of T cells among the clusters compares patients who developed cardiotoxicity with those who did not experience irAE. (E) Box plots illustrate the percentages of CD4 and CD8 T cell subsets among the different study groups. Statistical significance for unpaired comparisons was assessed using non-parametric -Kolmogorov-Smirnov t-test, assuming unequal SD. Data are presented as mean ± SD. The P-value is exact and two-tailed. (F&G) UMAP plots of CD8 subsets display the intensity of the T cell activation signature, with a background color intensity indicating cell density in relation to T cell activation intensity. A density plot compares the T cell activation score across patients with cardiotoxicity and no irAE, followed by a vertical bar plot comparing the T cell activation signature and type I and type II interferon response signatures among different T cell subsets. One-way ANOVA was used to determine the significance for the overall differences in scores across the different T cell subsets.

To explore peripheral T cell diversity in irAE cardiotoxicity, we initially conducted scRNA-seq on fifteen patients. After performing quality control, we analyzed 49,573 T cells from patients with cardiotoxicity and 13,386 T cells from control patients without irAEs. Longitudinal samples from three patients exhibiting cardiotoxicity included 14,666 pretreatment baseline and 13,810 treatment-phase cells. Figure 2A is a concatenated cluster of all single-cell RNA-seq data. Unsupervised clustering through a representative UMAP plot revealed 15 unique cell populations, with 10 showing CD3E expression (Fig. 2A). Six CD8 clusters were identified based on marker gene expression (Fig. 2B, Supp. Figure 2A) (Supp. Table 1). Cluster 2 consisted of naïve-like CD8 T cells, while cluster 11 exhibited characteristics of central memory. Cytotoxic effector phenotypes were found in clusters 3 and 5. Cluster 3 was characterized by granzyme A and B, together with perforin expression, and cluster 5 was characterized by Granzyme K along with Tbx21. Cluster 9 displayed early activation markers, indicating a transitional state, and cluster 4 showed a tissue-homing profile. We identified four CD4 T cell clusters (Fig. 2B, 2C): cluster 0 represented naïve CD4 T cells, cluster 1 exhibited T helper 2-like features, cluster 6 consisted of regulatory T cells, and cluster 10 displayed TH17-like traits. A comparative analysis showed notable differences in cluster distribution between cardiotoxicity patients and controls (Fig. 2D, Supp. Figure 2B). While the overall proportions of CD4 and CD8 T cells were similar (Supp. Figure 1C, 1D), specific subsets showed variations. FOXP3 + T cells were abundant in patients with cardiotoxicity, along with CD8 PRF1 + cytotoxic T cells significantly enriched compared to patient with no clinical signs of irAE (Fig. 2E). Moreover, CD8 TCF7 + T cells were prevalent in the baseline samples of patients who did not develop irAE, whereas CD4 TCF7 + cells were more common in those who did experience cardiotoxicity. Analysis of T cell activation scores revealed heightened activation of CD8 T cells across all subsets in cardiotoxicity patients (Fig. 2F; left) (Supp. Table 2). Type I interferon responses were sporadic (Fig. 2F; right), but CD8 type II interferon responses were significantly elevated in cardiotoxicity patients (Fig. 2F; right). A Type II interferon response was also elicited in CD4 T cells (Fig. 2H; right), specifically in FoxP3 + T cells, when comparing patients with cardiotoxicity and those with non-irAE.

#### Patients with irAE cardiotoxicity have increased levels of CD8 cytotoxic T cells at baseline.

We conducted a longitudinal analysis of five patients with sequential samples who experienced cardiotoxicity to assess any changes in T cell subset distribution before the cardiac events. Comparisons between baseline samples and those taken during the cardiotoxicity phase revealed an increase in CD8 PRF1 + cells in cluster 5 at baseline (Supp. Figure 3A-3C). Additionally, there was a notable rise in T cell activation and type II interferon signatures following ICI treatment (Supp. Figure 3B).

### Increased cytotoxicity of T cell clusters in irAE cardiotoxicity

**Figure 3:**
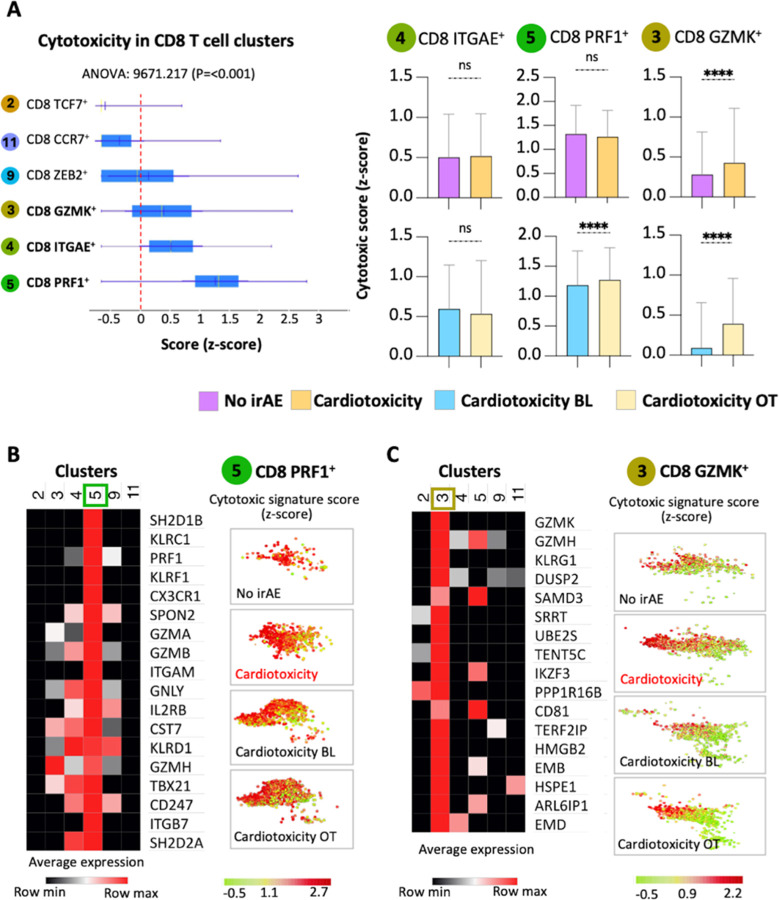
irAE cardiotoxicity T cell clusters are more cytotoxic. (A) Vertical bar plots illustrate the cytotoxic signature score across different CD8 T cell subsets, followed by horizontal bar plots that compare various conditions within those subsets. A heatmap displays the levels of RNA expression for dominant genes in cluster 5, corresponding to the CD8 PRF+ T cell subset (B) and the CD8 GZMK+ T cell subset (C). Additionally, a scatter plot representation shows the intensity of the cytotoxic signature score in the CD8 PRF+ or CD8 GZMK+ T cells among the different study groups. (C) The frequency of dots within the scatter plot reflects the proportion of CD8 PRF+ or CD8 GZMK+ T cells, with the color indicating the intensity of the cytotoxic signature score. The Mann-Whitney test assessed statistical significance for unpaired comparisons. Data are presented as mean ± SD, P-value, exact, two-tailed, * p < 0.05, ** p < 0.01, *** p < 0.001, **** p < 0.0001. One-way ANOVA was used to determine the significance for the overall differences in cytotoxicity scores across the different T cell subsets.

We analyzed gene expression profiles to gain insights into the functional state of expanded T cell clusters in patients experiencing cardiotoxicity. Cluster 5, which is rich in CD8 PRF1 + T cells, presented a distinct cytotoxic gene signature (Fig. 3A). Those with cardiotoxicity showed markedly elevated cytotoxic scores in this cluster compared to controls (Fig. 3B). Interestingly, cluster 3, identified by CD8 GZMK + T cells, also revealed increased cytotoxicity in both groups (Fig. 3C). Patients with cardiotoxicity had significantly higher cytotoxic scores in this cluster as well. No differences were observed in other clusters regarding gender, or among patients treated with monotherapy or combination therapy (Supp. Figure 4A-4D). These findings suggest that T cells in clusters 3 and 5 exhibit enhanced cytotoxic potential in individuals experiencing irAE cardiotoxicity.

### Distinct gene expression profile of CD8 PRF1 + and CD8 GZMK + T cells in irAE cardiotoxicity

**Figure 4:**
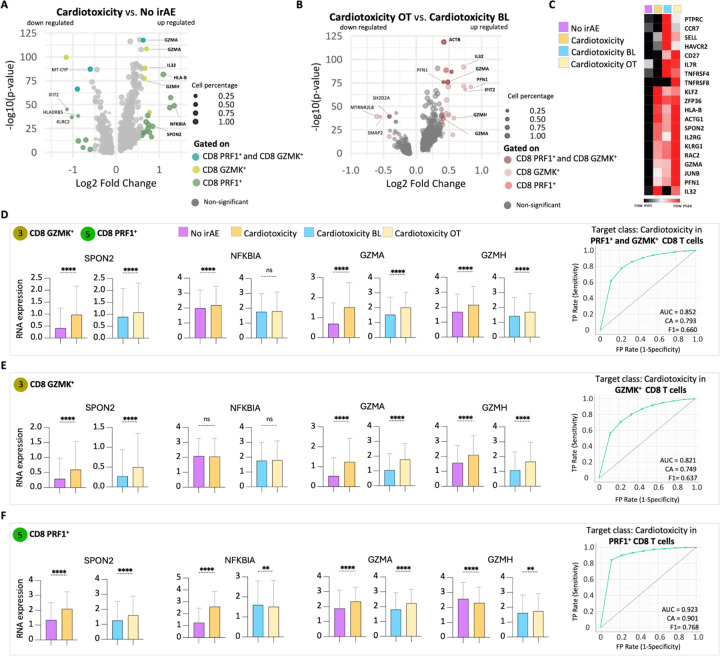
CD8 T cell subset of a patient with irAE cardiotoxicity expresses distinct genes. Volcano plots illustrating the genes that were either upregulated or downregulated in patients with cardiotoxicity compared to those with no irAE (A) and cardiotoxicity during treatment (OT) versus cardiotoxicity at baseline (BL) (B) in cluster 5 (CD8 PRF1+) or cluster 3 (CD8 GZMK+) or both clusters combined. (C) Heatmap displaying the levels of RNA expression of selective genes that were upregulated in patients with cardiotoxicity and during treatment (OT). Bar plots representing RNA expression levels of genes in the CD8 GZMK+ and CD8 PRF+ combined (D), CD8 GZMK+ (E), and CD8 PRF1+ T cell subsets (F), comparing the patients with cardiotoxicity and no-irAE. The Mann-Whitney test assessed statistical significance for unpaired comparisons. Data are presented as mean ± SD, P-value, exact, two-tailed, *p < 0.05, ** p < 0.01, *** p < 0.001, **** p < 0.0001. ROC curves differentiate patients who develop myocarditis based on gene transcript levels within the CD8 PRF+ T, CD8 GZMK+ T cell subset, or combined subsets.

To better understand how CD8 PRF1 + and CD8 GZMK + T cells contribute to cardiotoxicity, we conducted a detailed gene expression analysis (Fig. 4A). We found that several immune-related genes were significantly dysregulated in patients experiencing cardiotoxicity both during treatment and at baseline (Fig. 4B). This underscores their potential involvement in the development of the disease. Specifically, genes associated with T cell cytotoxicity and survival with the potential for cardiac injury, such as SPON2, GZMA, NFKBIA, and GZMH, exhibited increased activity in patients affected by cardiotoxicity, highlighting the significant role of CD8 PRF1 + and CD8 GZMK + T cells in the cardiac immune response (Fig. 4C). To explore the diagnostic potential of these gene expression profiles, we created receiver operating characteristic (ROC) curves (Fig. 4D–4F). The gene expression signatures, particularly the combined and individual GZMA levels in clusters 3 and 5 (Fig. 4D), demonstrated promising accuracy in predicting the development of cardiac toxicity.

### CD8 CD69 + T cells that express KLRF1 are involved in myocarditis

**Figure 5:**
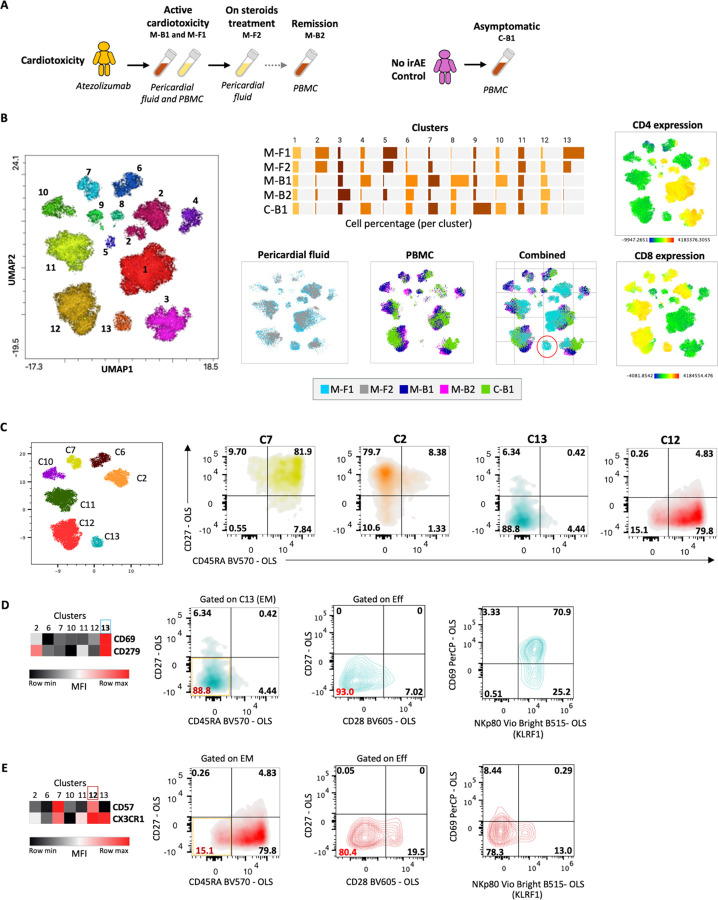
CD8 CD69+ T cells expressing KLRF1 are present in patients with myocarditis. (A) Blood and pericardial fluids were collected as specified. (B) UMAP plots demonstrate the distribution of cell clusters identified through flow cytometry. The split bar plot and UMAP plot represent the percentage and the distribution of cells in each cluster across various samples, and the UMAP intensity plot illustrates the expression of CD8 or CD4. (C) UMAP plot of only CD8+ clusters, along with the biaxial gating of different clusters using CD27 and CD45RA, is presented. (D) A heatmap shows the mean fluorescence intensity (MFI) of CD69 and PD-1 (CD279) among the clusters, followed by biaxial gating of cells from cluster 13 using CD45RA, CD27, CD28, NKp80 (KLRF1), and CD69. (E) Another heatmap presents the MFI of CD57 and CX3CR1 across clusters, along with bisecting gating on cells from cluster 12 using CD45RA, CD27, CD28, NKp80 (KLRF1), and CD69.

To validate our findings from scRNA-seq at the protein level, we examined blood and pericardial fluid from myocarditis patients and a non-irAE control using 38-marker spectral flow cytometry (Fig. 5A). Through UMAP analysis, we identified 13 clusters, including seven CD8 and five CD4 T cell populations (Fig. 5B). By integrating UMAP with traditional biaxial gating, we distinguished four CD8 T cell subsets in each cluster based on CD27 and CD45RA expression (Supp. Figure 5A-5C). Cluster 13, enriched in pericardial fluid, mainly comprises CD69 + PD-1 + effector CD8 T cells (Fig. 5C, 5D). While cluster 12 also had effector CD8 T cells, it showed lower CD69 and KLRF1 expression than cluster 13 (Fig. 5D, 5E). This finding was further validated by the expression of CD69 and CX3CR1 in cluster 13, accounting for a total of 31% of the cells present in the pericardial fluid of myocarditis patients, compared to 1.08% and 0% of cells present in the PBMCs of matched myocarditis or no irAE patients, respectively (Supp. Figure 5Di). In contrast, CD69-low effector CD8 cells that were positive for CX3CR1, found within cluster 12, were more enriched in PBMCs than in pericardial fluid (Supp. Figure 5Dii). Additionally, all CD69 + effector CD8 cells in cluster 13 did not express KLRG1 (Supp. Figure 5E). These data suggest that CD69 + effector CD8 T cells, predominantly expressing PD-1 (CD279) and KLRF1 in cluster 13, may be implicated as immune effectors in the development of myocarditis or may act as its potential mediators.

#### Cardiotoxicity TCR CDR3 clones exhibit greater hydrophobicity.

**Figure 6:**
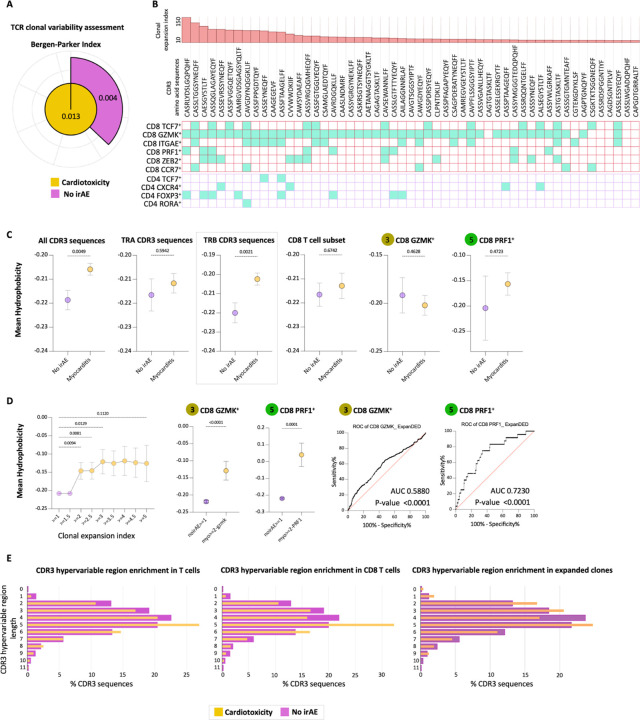
Cardiotoxicity TCR CDR3 clones exhibit greater hydrophobicity. (A) The Bergen-Parker index provides values for various patient groups. (B) Heatmap-bar plot representing the distribution of specific CDR3 amino acid sequences (termed “clones”) among different CD8+ and CD4+ T cell subsets, arranged by decreasing order of clonal expansion index (the ratio of cardiotoxicity to non-irAE dominant clone, multiplied by 100). (C) Dot plots illustrate the differences in mean hydrophobicity among various comparison groups. (D) Dot plots depict the mean hydrophobicity differences correlating with clonal expansion for all expanded clones, particularly those in the CD8+ GZMK+ and CD8+ PRF+ T cell subsets. Statistical significance was assessed using the Kruskal-Wallis ANOVA test. ROC curves were generated to distinguish cardiotoxicity-associated expanded clones from non-expanded clones within the CD8+ PRF+ T cell subset. (E) The overlapped bar plot illustrates the enrichment of the CDR3 hypervariable region in relation to the length of the CDR3 mid-hypervariable region, comparing cardiotoxicity to non-irAE. The Mann-Whitney test was used to determine statistical significance in unpaired comparisons. Data are presented as mean ± SD, with exact two-tailed P-values.

To explore the dynamics of TCR repertoires, we conducted scTCR-seq analysis on 53,420 cells with productive TCRs. Patients with cardiotoxicity showed greater TCR diversity and clonal expansion compared to control subjects (Fig. 6A). The expanded clones were prevalent in both CD8 and CD4 T cell subsets, especially among CD8 GZMK + and CD8 PRF1 + populations (Fig. 6B). Certain B-chains CDR3 regions were shared between different phenotypic subsets and across CD4 and CD8 subsets, indicating promiscuity (23). Notably, these cardiotoxicity patients had higher CDR3 hydrophobicity, particularly in TRB chains (Fig. 6C). This pattern was also significant in expanded CD8 PRF1 + clones, correlating with the degree of the clonal expansion (Fig. 6D). The total CDR3 length distribution of all CD8 clones was longer in the cardiotoxicity group; however, the expanded clones (Fig. 6E; right) exhibited a bimodal distribution, showing a preference for expanded short CDR3 clones. These results suggest that more hydrophobic, shorter CDR3 sequences in expanded CD8 T cell clones may play a role in the pathogenesis of irAE cardiotoxicity.

### Analysis of cardiac tissue T cells

**Figure 7:**
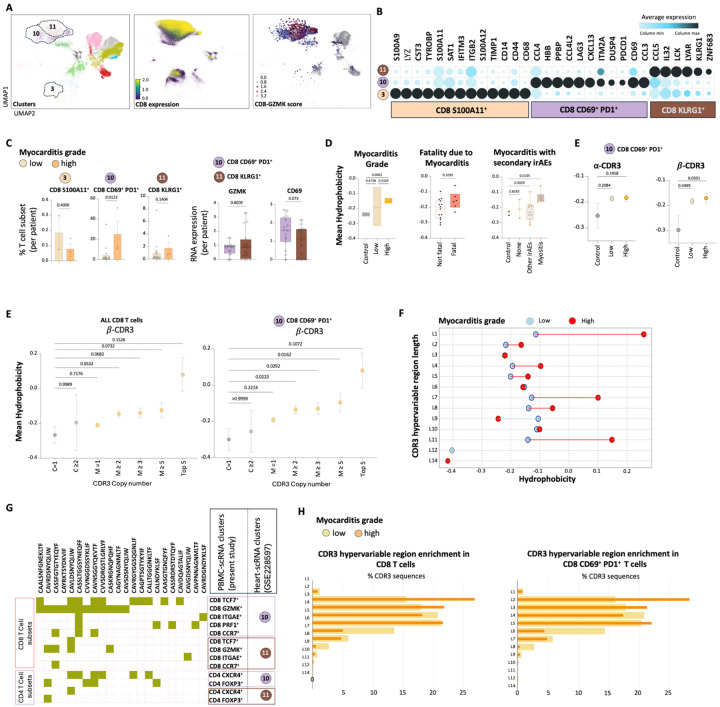
Analysis of cardiac tissue T cells. (A) UMAP plots based on scRNA sequencing of cells harvested from heart tissue (dataset GSE228597). Clusters 3, 10, and 11, outlined, represent CD8 GZMK+ T cells, as indicated by CD8 expression and GZMK signature score. (B) Marker gene expression levels differentiate CD8 T cell clusters 3, 10, and 11. (C) Bar plots illustrate the percentages of CD8 T cell subsets among patients with either low- or high-grade myocarditis. Statistical significance for unpaired comparisons was assessed using the Mann–Whitney test. Data are presented as mean ± SEM. The p-value is precise and two-tailed. Box plots display RNA expression levels of GZMK and CD69 genes per patient in cluster 10 (CD8 CD69+ PD1+) and cluster 11 (CD8 KLRG1+). (D) Floating bar and box plots represent the differences in mean hydrophobicity computed for the CDR3 mid-hypervariable region across different comparison groups. (E) Dot plots depict the differences in mean hydrophobicity computed from either combined β-CDR3 sequences (mid-region) or β-CDR3 sequences (mid-region) for TCR clones representing cluster 10 (CD8 CD69+ PD1+). Using the Brown-Forsythe ANOVA test, these dot plots illustrate mean hydrophobicity differences correlated with increasing CDR3 copy number in all CD8 T cells or only in cluster 10 (CD8 CD69+ PD1+ T cells). (F) Range plots indicate the hydrophobicity differences in the CDR3 hypervariable region arranged by the length of the CDR3 mid-hypervariable region, comparing sequences from low- and high-grade myocarditis patients. (G) Heatmap plots represent the distribution of specific CDR3 amino acid sequences among CD8 and CD4 T cell subsets shared between the PBMC dataset (the present study) and the heart tissue dataset. (H) The overlapped bar plot demonstrates the enrichment of the CDR3 hypervariable region organized by the length of the CDR3 mid-hypervariable region, comparing sequences from low- and high-grade myocarditis in CD8 T cells and the subset CD8 CD69+ PD1+ T cells.

To validate our findings using an alternative source of irAE myocarditis samples, we analyzed data from Blum et al., focusing on the heart tissue biopsies (14). This study examined T cells sourced from the heart tissue of patients with ICI myocarditis, focusing on scRNA and TCR sequencing. To correlate with our previous findings (Fig. 3), we re-analyzed the dataset, employing the dimensional reduction - UMAP plot to eventually identify three CD8 T cell clusters expressing GZMK, in addition to other less relevant clusters (Fig. 7A). These clusters were annotated as cluster 3 (CD8 S100A11+), cluster 10 (CD8 CD69 + PD1+), and cluster 11 (CD8 KLRG1+) (Fig. 7B). Because the number of T cells from the control heart tissue was too low for a fair comparison, we categorized the patients into two groups based on the clinical severity of myocarditis: high grade and low grade. We then compared the cellular characteristics between these groups. Similar to what we showed in the peripheral blood (Fig. 3 and Fig. 4), both cluster 10 and cluster 11 express GZMK (Fig. 7C). Notably, cluster 10 was more prevalent in patients exhibiting high-grade myocarditis (Fig. 7C) and, similar to our initial data, this cluster also showed a high level of CD69 expression (Fig. 5D).

Cells from patients with high-grade myocarditis, fatal outcomes, and concurrent myositis also presented a higher hydrophobicity score (Fig. 7D). Notably, the beta chains of CDR3 regions exhibited significantly greater hydrophobicity in myocarditis patients compared to healthy controls, surpassing the increase observed in the alpha chain alone or in combination with the beta chain. Additionally, there was a noticeable sharing of expanding CDR3 sequences between our PBMC dataset and heart tissue dataset, largely absent in control or no-irAE patients (Fig. 7E, Supp. Figure 6). To further establish hydrophobicity as a key determinant, we observed a proportional increase in CDR3 hydrophobicity with the expansion of T cell clones, consistent with the pattern identified in the initial cohort (Fig. 7E). Hydrophobicity was consistently higher across all mid-hypervariable CDR3 lengths in high-grade myocarditis compared to low-grade myocarditis, indicating a potential association between CDR3 length, hydrophobicity, and disease severity (Fig. 7F). Notably, CDR3 sequences enriched in the peripheral blood of cardiotoxicity patients were found within the same phenotypic subset as those enriched in the myocarditis dataset. CDR3 sequences associated with the CD8 CD69 + PD-1 + T cell subset (cluster 10; Fig. 7), characterized by elevated GZMK expression, were also identified in the CD8 GZMK + subset of cardiotoxicity patients (cluster 3; Fig. 2). Furthermore, these CDR3 sequences were shared across T cell subsets, including both CD8 and CD4 populations, indicating potential cross-lineage clonal relationships and functional plasticity (Fig. 7G, Supp. Figure 7). As highlighted in our peripheral blood TCR analysis, CDR3 sequences from high-grade myocarditis exhibited a shorter, bimodal distribution compared to those from low-grade myocarditis. (Fig. 7H). These data validate our earlier finding that the pathogenic CD8 effector T cells in irAE myocarditis have shorter and more hydrophobic CDR3 sequences.

### Codon redundancy and TCR clonal expansion in irAE cardiotoxicity

**Figure 8:**
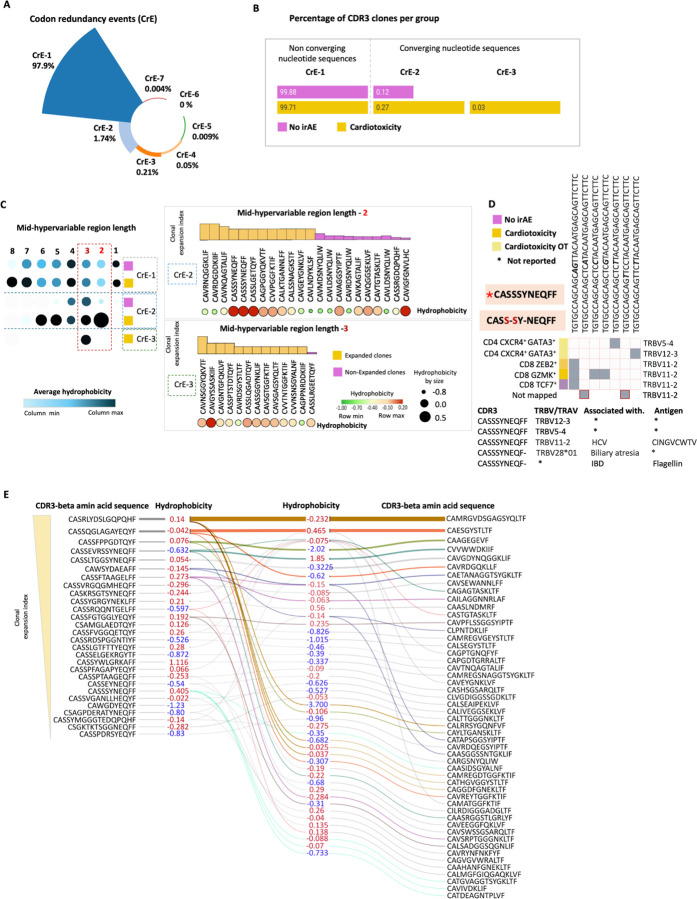
TCR clones of irAE cardiotoxicity patients show higher codon redundancy events clonal convergence. (A) A circular bar plot displays the percentage distribution of codon redundancy events, representing the count of CDR3 nucleotide sequences that converge to form identical CDR3 amino acid sequences in all CD3+ T cells. (B) Split bar plots illustrate the percentage differences in the enrichment of non-converging and converging clones in patients without irAE and those with cardiotoxicity. (C) A dot-plot heatmap presents the mean hydrophobicity comparison between converging sequences (CrE-2 and CrE-3) and a non-converging sequence (CrE-1) using the mid-hypervariable length of the CDR3 amino acid sequence. The enriched CDR3 clones within the CrE2 and CrE3 groups are depicted as bar plots arranged from high to low clonal expansion index. At the same time, the associated hydrophobicity score is shown as a dot-heatmap. (D) An example of a converging clone is provided, demonstrating shared TRBV elements across various T cell subsets. (E) Alluvial plot depicting the correlation between hydrophobicity and preferential bias between in CDR3-beta and CDR3-alpha sequences of expanded clones.

Codon redundancy, or degeneracy, of the genetic code refers to the phenomenon where multiple codons can encode the same amino acid during protein synthesis. This phenomenon has been observed in autoimmune diseases, where clonal selection occurs at the protein level, rather than the nucleotide level. Despite the high diversity of TCRs, a notable increase in codon redundancy events (CrE) was observed in patients with cardiotoxicity relative to controls (Fig. 8A, 8B). CrE appeared to correlate with heightened hydrophobicity and reduced CDR3 lengths, particularly in expanded clones from patients experiencing cardiotoxicity (Fig. 8C). Analyzing V gene segment usage revealed a relationship between codon redundancy and TCR recombination. Various nucleotide sequences can yield a singular CDR3 amino acid sequence through different V gene segments (Fig. 8D, Supp. Figure 7A). Additionally, multiple CDR3-alpha chains could pair with CDR3-beta chains, further highlighting the intricate nature of the TCR repertoire among cardiotoxicity patients (Fig. 8E, Supp. Figure 7A). These results suggest that codon redundancy and V gene segment usage contribute to TCR clonal convergence, with the CDR3 mid-hypervariable region populated by hydrophobic residues, potentially leading to the development of ICI-associated cardiac irAE.

### Hydrophobic CDR3β region and MHC-I interactions in irAE cardiotoxicity

**Figure 9:**
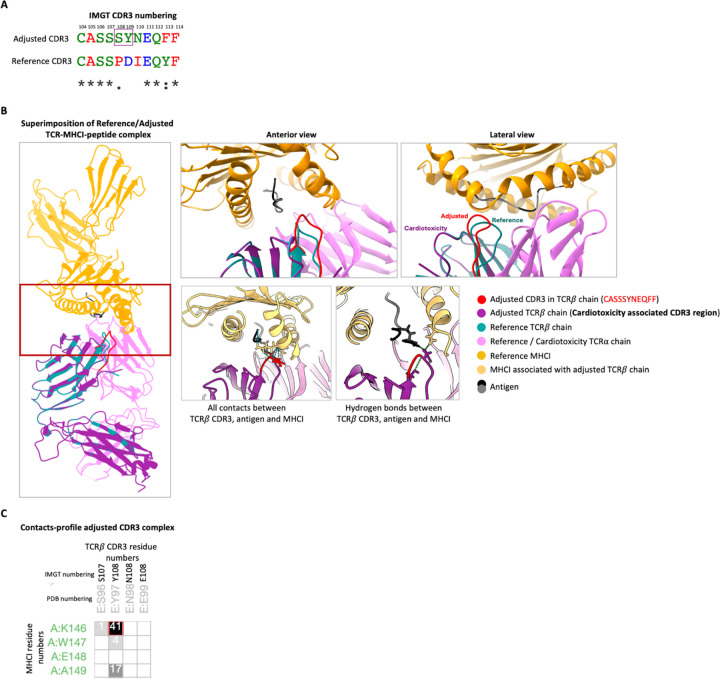
CDR3b of irAE cardiotoxicity may interact with MHCI independent of peptide influence. (A) The sequence alignment of reference CDR3-beta residues with cardiotoxicity-related CDR3-beta is performed using the TCR3d web portal, presented in the IMGT numbering format. Asterisks indicate 100% identity among residues. The mid-hypervariable region is highlighted in a purple box. (B) The simulated structures of 7N1F-Reference and 7N1F-Adjusted are superimposed and depicted as a cartoon representation of the TCR-pMHCI complex using ChimeraX. This section includes an anterior and lateral view that showcases the conformational displacement of the CDR3-beta, with red color illustrating the adjusted CDR3 loop, teal color denoting the reference CDR3 loop, and purple color representing cardiotoxicity associated TCR-beta chain. An anterior view highlights the CDR3-MHCI interaction through total contacts and hydrogen bonds involving the adjusted mid-hypervariable region of the 7N1F-Adjusted structure. (C) A profile of the interactions between adjusted TCR-β CDR3 residues and MHCI residues is presented.

We performed structural analyses using the Maestro-Schrodinger package to investigate how CDR3 length and hydrophobicity may influence TCR-MHCI interactions. A reference TCR-MHCI-peptide complex (7N1F) was modified to incorporate a cardiotoxicity-associated CDR3β sequence, focusing on the mid-hypervariable region. We conducted molecular dynamics simulations on both the reference and modified structures confirmed their lower energy state and stabilization depicted through the Ramachandran plot (Supp. Figure 7B). Although the CDR3β region retained conserved residues, the mutated CDR3β loop displayed an outward conformational shift, bringing it nearer to the MHCI chain (Fig. 9B). This adjustment allowed for new hydrophobic interactions and hydrogen bonds between the mutated CDR3β and MHCI residues (Fig. 9C), which are absent in the reference structure (15). These results suggest that the hydrophobic properties of the modified CDR3β region might influence TCR-MHCI interactions more than TCR-peptide-MHC interactions, potentially impacting T cell recognition and activation in irAE cardiotoxicity.

### CDR3 sequences from patients with irAE arthritis are shorter and more hydrophobic

To further validate our findings using a different organ irAE, we analyzed scRNA and scTCR sequencing data of T cells collected from cancer patients treated with ICI who developed inflammatory arthritis (GSE216329) (26). KNetL projection identified 25 T cell clusters (Supp. Figure 8A). TCR clonal analysis revealed a higher fold increase in clonal expansion in patients with irAE arthritis compared to those without irAE (Supp. Figure 8B). The highest expansion originated from clones in CD8 TE TBX21+ (cluster 16) expressing high levels of PRF1, and EOMES (cluster 19), which expressed elevated levels of GZMK, similar to the expanded T cell clones observed in myocarditis patients (Fig. 4, Supp. Figure 8C-8D). Furthermore, CDR3s from expanded T cell clones of patients with arthritis were more hydrophobic than CDR3s from patients without irAE (Supp. Figure 8E). CD8 RFP1 + T cells from the patient with arthritis also demonstrated a higher hydrophobic score compared to T cells from patients with no irAE (Supp. Figure 8F). The frequency distribution plot revealed a correlation between hydrophobicity and the copy number of expanded CD8 TE TBX21 + PRF1 + T cell clones (Supp. Figure 8G). A ROC curve utilizing hydrophobic values of arthritis-enriched T cell clones revealed an area of 0.74 and a p-value of 0.0004 (Supp. Figure 8H). Superimposed simulated structures of no-irAE and arthritis-associated CDR3 inserts into TCR-p-MHC complexes supported the findings from the myocarditis dataset, revealing that hydrophobicity plays a key role in positioning the CDR3β loop in closer proximity to MHCI residues (Supp. Figure 8I).

## DISCUSSION

ICI-induced cardiotoxicity represents a severe and occasionally fatal adverse effect observed in patients undergoing either immuno-monotherapy or combined immunotherapy (16–19). In the present study, patients who developed myocarditis subsequent to ICI treatment exhibited a notable increase in circulating cytotoxic perforin-secreting T cells (CD8 + PRF1 + T cells), which coincided with the onset of the disease. Elevated levels of these effector CD8 T cells were also identified in the cardiac tissue and pericardial fluid of patients with myocarditis, suggesting their potential role in exacerbating myocardial injury. Of particular significance, at baseline, patients who manifested irAEs with myocarditis were characterized by considerably higher concentrations of CD8 PRF1 + T cells compared to those who did not experience myocarditis. These T cells predominantly shared their TCR clonotype with the additionally expanded clones present in myocarditis patients, indicating an immune response that may predispose to the development of ICI-induced cardiotoxicity. Conversely, patients devoid of cardiotoxicity exhibited increased baseline levels of CD8 TCF7 + T cells, which were observed to decline following ICI treatment. Notably, the expanded CD8 PERF1 + T cell clones from patients exhibiting irAE myocarditis were distinguished by bimodal and shorter TCR CDR3-chain sequences, which contained significantly higher proportions of more hydrophobic amino acid residues. As discussed below, this observation indicates a novel mechanistic role through enhanced TCR engagement and activation in irAEs (20, 21).

Cytotoxic CD8 T cells, including PRF1 + and GZMK + cells, are known to be involved in the pathogenesis of autoimmune diseases and viral myocarditis. However, limited data show their role in irAE cardiotoxicity. CD8 T cells that target the cardiac protein α-myosin heavy chain have been shown to play a crucial role in the development of myocarditis associated with ICI therapy. In mouse models that simulate ICI-related myocarditis, these cells infiltrate the heart and play a vital role in the disease’s progression (27). Additionally, cytotoxic CD8 T cells cause myocardial damage in patients suffering from virus-induced myocarditis (28). Upon activation, CD8 T cells can directly kill cardiomyocytes through contact mechanisms, such as Fas ligand/receptor interactions, and by releasing toxic compounds, including perforin, granzymes, and granulysin. They also contribute to inflammation by producing pro-inflammatory cytokines, such as TNF-α and IFN-γ. Furthermore, they may play a role in myocardial pathology through non-antigen-specific mechanisms, supporting the model we proposed in Fig. 9 (29). It is essential to identify CD8 cytotoxic T cells involved in the pathogenesis of irAE myocarditis, as this indicates that targeting the PAR1 signaling pathway could be an effective therapeutic strategy for treating irAE cardiotoxicity (30).

The existence of hydrophobic CDR3 TCR clones, along with their notable clonal expansion, underscores the importance of these T cell populations in cardiac injury (22). The relationship between TCR hydrophobicity and autoimmune diseases is an area of ongoing research, but it has not been discussed in the context of ICI therapy or irAE pathogenesis. Some studies have indicated that self-reactive CD4 and CD8 T cells may exhibit an increased presence of hydrophobic residues, particularly in their CDR3 regions (31). Cysteine and hydrophobic residues in the CDR3 have been suggested as potential indicators of T cell self-reactivity (32). Research has established a link between TCR hydrophobicity and autoimmune diseases, revealing a connection to Wiskott-Aldrich Syndrome (WAS), in which 24–72% of patients develop autoimmune conditions (33). More specifically, these studies have demonstrated a preference for hydrophobic amino acids at TCR contact residues within immunogenic epitopes on MHC molecules. This finding has spurred the creation of hydrophobicity-based models to predict immunogenic epitopes, which may aid in understanding and treating autoimmune diseases. Although some evidence suggests a connection, further extensive studies are needed to fully understand the impact of TCR hydrophobicity in other autoimmune diseases. Additional research, potentially incorporating advanced methods such as TCR sequencing and computational analysis, is essential to predict the convergent features of pathogenic TCRs in irAE disorders.

Our findings indicate a notable rise in type II interferon responses among patients with cardiotoxicity. This points to a possible involvement of type II interferon-mediated immune responses in the development of ICI-related cardiotoxicity. Type II Interferon (IFN-γ) plays a critical role in antiviral immune responses and autoimmune disorders. While type I interferons are primarily linked to the immediate antiviral response, IFN-γ is vital for establishing and sustaining the adaptive immune response, also playing a part in the onset and progression of autoimmune diseases. Specifically, IFN-γ is associated with systemic lupus erythematosus (SLE) where it may contribute to the formation of autoantibodies and lead to tissue damage (34). Although IFN-γ is crucial for combating infections, its overactivation or dysregulation can promote autoimmunity. In certain autoimmune diseases, IFN-γ facilitates the emergence of autoreactive B cells that create autoantibodies targeting self-antigens. Thus, the role of IFN-γ in autoimmunity indicates that targeting its signaling pathways may offer a potential therapeutic approach for specific autoimmune conditions (35). Since the treatment approach to irAE, in some cases, resembles that of primary autoimmune diseases, it is crucial to exclude treatments like anifrolumab, which is designed to target type I immune responses.

Our sequencing data revealed unique traits in the TCR repertoire of patients with irAE cardiotoxicity. Compared to controls, patients exhibited a higher number of expanded TCR clones, particularly in the CD8 GZMK + and CD8 PRF1 + subsets. These clones exhibited increased hydrophobicity, a bimodal distribution, and, in some cases, shorter CDR3 hypervariable regions. A strong correlation between CDR3 hydrophobicity and clonal expansion suggests a potential link to pathogenic T cell activity (22–23). Additionally, the observed preferential pairing between TRBV usage and CDR3 beta chains in expanded clones may contribute to aberrant TCR signaling. More specifically, our data reveal a distinct pattern of TRBV usage and CDR3 pairing (Fig. 7E, Supp. Figure 7A), with expanded T cell clones displaying shared characteristics in their CDR3 regions, notably shorter and more hydrophobic sequences. Furthermore, shorter and hydrophobic CDR3 beta sequences are also preferentially paired with shorter CDR3 alpha sequences, facilitated by convergent TRBV usage. This structural variation could enhance T cell activation in the context of potential peptide-independent TCR-MHC interactions. Such activation could underlie the dysregulation of immune responses and contribute to the development of irAE. It also indicates an existing immune response that selectively utilizes structurally unique T cells.

It is noteworthy to observe (Fig. 7H) that we identified two distinct populations of expanded T cells. One population possesses a shorter CDR3 at 12, while the other exhibits a longer TCR at 15. Although the longer T cells hold significance in their own right, they are likely to function through conventional mechanisms. In contrast, the shorter T cells may represent the peptide non-specific variety, which adds an intriguing dimension to our findings. Our research suggests that shorter, hydrophobic CDR3 sequences in expanded T cell clones may facilitate the activation of these cells in a pathological context, independent of traditional peptide-MHC interactions (24, 25). Our structural analysis (Fig. 9) corroborates this hypothesis, suggesting that the hydrophobic regions of CDR3 can directly engage with the MHC-I molecule, thereby obviating the requirement for peptide stabilization. This recognition mechanism of T cells may contribute to the clonal expansion and pathogenicity observed in ICI-related cardiotoxicity, specifically including myocarditis.

A recent study by Blum et al. (14) offers key insights into myocarditis induced by ICI, deepening our understanding of its immunopathology. This research investigates immune environments in advanced myocarditis by analyzing paired samples from the heart, blood, and tumors, thus highlighting localized immune interactions. While the study outlines the relationships among cytotoxic T cells, dendritic cells, and fibroblasts within myocarditis-affected hearts, our findings expand the discussion by linking these mechanisms to baseline immune profiles. Our re-examination of the myocarditis heart biopsy dataset from Blum et al. revealed a notable overlap of enriched TCR clones with those identified in T cells, emphasizing the potential for using minimally invasive blood samples for early detection and mechanistic studies. Our research highlights the predictive and mechanistic value of baseline PBMC profiling, uncovering immune signatures in patients at risk of cardiotoxicity. We revealed important cellular mechanisms contributing to this predisposition through detailed TCR repertoire and T cell subset investigations, including the clonal expansion of hydrophobic CDR3 sequences predominantly in CD8 GZMK + T cells and, to a lesser extent, in CD8 PRF1 + T cells.

### Limitations

Despite some acknowledged limitations, this analysis provides valuable insights into immune-related cardiotoxicity associated with ICIs. Nonetheless, the inclusion of an imaging study would enhance the research. Cardiac MRI data were limited to only a few patients, creating uncertainty in diagnosing confirmed myocarditis. To validate our conclusions, guide risk management strategies for vulnerable patients, and assist in developing targeted therapies and biologically informed treatments for ICI-induced myocarditis, it is crucial to conduct analyses that utilize larger datasets with accessible tissue samples or prospective studies that involve longitudinal sampling. Future research should elucidate the role of these T cell subsets in myocardial injury, with the potential to profile these characteristics and identify individuals at risk. This could also reveal opportunities to target these cells or related pathways to mitigate toxicity.

## Conclusions

Our research elucidates the cellular and structural characteristics of the immune landscape associated with ICI cardiotoxicity. By elucidating critical T cell subsets and their molecular features, we have identified clinical screening strategies to ascertain at-risk patients and enhance their treatment. We have also laid the groundwork for innovative therapies to mitigate this adverse effect. Through baseline immune profiling and hydrophobic TCR traits, we aim to develop interventions that reduce the risk of myocarditis while maintaining the antitumor efficacy of ICIs, ultimately bridging cancer immunotherapy and immune-related safety.

## Supplementary Material

Supplementary Files

This is a list of supplementary files associated with this preprint. Click to download.

- Supplementarytable1copy.xlsx
- Supp.Figure1.tiff
- Supp.Figure1.tiff
- Supp.Figure3.tiff
- Supp.Figure4.tiff
- Supp.Figure5.tiff
- Supp.Figure6.tiff
- Supp.Figure7.tiff
- Supp.Figure8.tiff
- Supp.Figure8Legend.tiff

## Data Availability

The data analyzed in this study are subject to the following licenses/restrictions: Qualified researchers may request access to individual patient-level data for each separate study through a data request platform. The data sets are only available as individual data sets per study and are not integrated across studies. For up-to-date details on Roche’s Global Policy on the Sharing of Clinical Information and how to request access to related clinical study documents, see here: https://go.roche.com/data_sharing. Anonymized records for individual patients across more than one data source external to Roche cannot, and should not, be linked due to a potential increase in risk of patient re-identification. Requests to access these data sets should be directed to am5121@cumc.edu.
